# FET fusion oncoproteins disrupt physiologic DNA repair networks and induce ATR synthetic lethality in cancer

**DOI:** 10.21203/rs.3.rs-2869150/v1

**Published:** 2023-05-29

**Authors:** Shruti Menon, Marcus R. Breese, Yone Phar Lin, Hannah Allegakoen, Shruthi Perati, Ann Heslin, Max A. Horlbeck, Jonathan Weissman, E. Alejandro Sweet-Cordero, Trever G. Bivona, Asmin Tulpule

**Affiliations:** 1 Tow Center for Developmental Oncology and Human Oncology and Pathogenesis Program, Memorial Sloan Kettering Cancer Center, 1275 York Avenue, New York, NY, 10021.; 2 Department of Pediatrics, Memorial Sloan Kettering Cancer Center, 444 East 68th Street, 9th Floor, New York, NY 10065.; 3 Division of Pediatric Oncology, University of California, San Francisco, San Francisco, CA 94143.; 4 Division of Genetics and Genomics, Boston Children’s Hospital, Boston, MA, 02115.; 5 Department of Biology, Massachusetts Institute of Technology, 77 Massachusetts Ave, 68-132, Cambridge, MA 02139.; 6 Division of Hematology and Oncology, University of California, San Francisco, San Francisco, CA 94143.

## Abstract

The genetic principle of synthetic lethality is clinically validated in cancers with loss of specific DNA damage response (DDR) pathway genes (i.e. BRCA1/2 tumor suppressor mutations). The broader question of whether and how oncogenes create tumor-specific vulnerabilities within DDR networks remains unanswered. Native FET protein family members are among the earliest proteins recruited to DNA double-strand breaks (DSBs) during the DDR, though the function of both native FET proteins and FET fusion oncoproteins in DSB repair remains poorly defined. Here we focus on Ewing sarcoma (ES), an EWS-FLI1 fusion oncoprotein-driven pediatric bone tumor, as a model for FET rearranged cancers. We discover that the EWS-FLI1 fusion oncoprotein is recruited to DNA DSBs and interferes with native EWS function in activating the DNA damage sensor ATM. Using preclinical mechanistic approaches and clinical datasets, we establish functional ATM deficiency as a principal DNA repair defect in ES and the compensatory ATR signaling axis as a collateral dependency and therapeutic target in FET rearranged cancers. Thus, aberrant recruitment of a fusion oncoprotein to sites of DNA damage can disrupt normal DSB repair, revealing a mechanism for how oncogenes can create cancer-specific synthetic lethality within DDR networks.

The DNA damage response (DDR) is a tightly regulated and redundant network that tailors specific repair complexes to address a diverse set of genotoxic insults. In cancer, genetic loss of certain DDR components creates tumor-specific vulnerabilities that can be therapeutically exploited^1^. The canonical example of DDR synthetic lethality is the use of poly-ADP ribose polymerase (PARP) inhibitors to target defective homologous recombination (HR) repair in BRCA1/2 mutant breast and ovarian cancers^2,3^. The broader applicability of DDR-directed therapies in cancers without genetic DDR alterations and whether oncogenes create vulnerabilities within DDR networks remains to be defined.

The FET family of intrinsically disordered proteins (FUS, EWS, TAF15) are frequent 5’ oncogenic transcription factor (TF) fusion partners in a diversity of sarcomas and leukemias^4^. The most studied cancer in this class is Ewing sarcoma (ES), a pediatric bone tumor driven by the EWS-FLI1 TF fusion oncoprotein. Patients with relapsed or metastatic ES continue to have dismal outcomes despite maximally intense combination chemotherapy regimens^5^. Paradoxically, decades of clinical experience and laboratory testing of ES cancer cell lines have shown ES to be among the most chemo- and radiosensitive cancers^6–9^, at least initially. It has long been hypothesized that ES tumors harbor a DNA repair defect that explains their underlying sensitivity to DNA damaging therapies. The prevailing model is that ES belongs to a family of “BRCA-like” tumors that are functionally deficient in BRCA1 (due to sequestration of BRCA1 protein by RNA-DNA hybrid structures known as R-loops) and therefore defective in HR double-strand break (DSB) repair^10^. However, both xenograft studies^8^ and clinical trials in ES patients^11^ failed to demonstrate any benefit for PARP inhibitor monotherapy, in stark contrast to the impressive clinical responses seen across HR deficient BRCA mutant and BRCA-like cancers^12,13^. Thus, the precise nature of the DNA repair defect in ES remains uncertain.

Native FET family proteins contain an N-terminal intrinsically disordered region (IDR) required for interactions amongst FET family members and a C-terminal domain with positively charged RGG (arginine-glycine-glycine) repeats, which mediate recruitment to DSBs via high affinity interactions with negatively charged poly-ADP ribose (PAR) molecules^14,15^. All 3 FET members are rapidly recruited to DSBs in a PARP-dependent manner^15,16^, where they undergo liquid-liquid phase separation that is thought to enable compartmentalization of DSB repair proteins^17,18^, though the specific role of FET proteins in DSB repair is not well defined. Interestingly, all oncogenic FET fusion proteins including EWS-FLI1 share a similar structure: the N-terminal IDR of the FET protein fused to the DNA binding domain of a transcription factor (e.g. FLI1), with loss of the C-terminal RGG repeats^19^.

In this study, we address the role of the oncogenic fusion protein EWS-FLI1 in regulating the DNA damage response in ES. Contrary to the current classification of ES as a BRCA-like tumor with defective HR^10^, we identify functional ATM deficiency as the principal DDR lesion in ES cells and define the mechanistic basis for EWS-FLI1 mediated DNA repair defects in ES. More broadly, our findings demonstrate how an oncoprotein can create tumor-specific vulnerabilities within the DDR network.

## Results

### ES cells are dependent on HR factors for survival.

**Figure 1:**
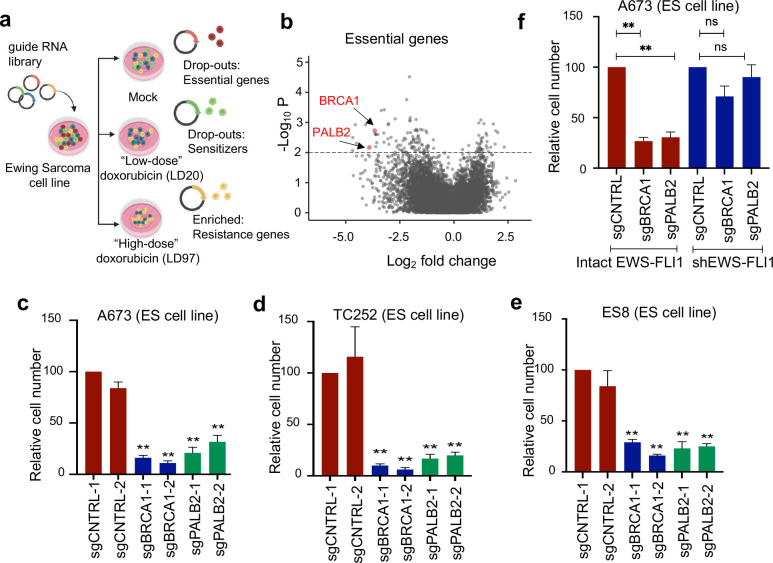
ES cells are dependent on HR factors for survival. A) Schematic of CRISPRi genetic screen of 7,000 cancer associated genes in ES cell line A673. B) Volcano plot of sgRNA log_2_fold change (X-axis) vs −log10(p value), average of 2 biologic replicates. Full screen results in Supplementary Table 1. C-E) Growth assays in dCas9 expressing ES cell lines upon introduction of 2 independent sgRNAs against BRCA1, PALB2, or control. n = 4. F) Growth assays in ES cell line A673 with doxycycline-inducible shRNA against EWS-FLI1. For all panels, error bars represent ± SEM, **p < 0.01 by one-way ANOVA with post hoc Tukey’s HSD test.

To address the uncertainty surrounding putative DNA repair defect(s) in ES, we set out to identify genes that modulate ES cell survival in response to doxorubicin, a DNA DSB-inducing agent and major component of current chemotherapy regimens^20^ (Fig. 1A). We selected a CRISPR interference (CRISPRi) based screening approach given the concern of studying DSB repair phenotypes using an active Cas9 that generates DSBs^21^. Surprisingly, we found that key HR factors BRCA1 and PALB2 were essential for the growth of ES cells even in the absence of doxorubicin (Fig. 1B). Given the prevailing model that ES tumors are functionally HR-deficient, BRCA-like tumors^10^, the screen results were unexpected. We validated the finding of HR factor dependency in ES using two independent guide RNAs (gRNAs) against BRCA1 and PALB2 in the screening cell line (A673) and two additional ES cell lines TC71 and ES8 (Figs. 1C–E and S1A, B). In contrast, the same BRCA1 and PALB2 gRNAs had limited effects on cell growth in two non-ES cancer cell lines, which was consistent with the set of published CRISPRi screens^21–23^ and suggested the observed dependency on HR factors may be specific to ES cells (Figs. S1C, D). To test the role of EWS-FLI1 in inducing this dependency, we utilized an ES cell line with a doxycycline-inducible shRNA against EWS-FLI1^24^ (Fig. S1E). We observed that EWS-FLI1 knockdown rescued the growth defects caused by BRCA1 or PALB2 loss, confirming that the oncogenic fusion protein is necessary for the observed dependency on HR factors in ES cells (Fig. 1F).

CRISPRi screening also identified genes whose loss sensitized ES cells to doxorubicin including LIG4, NHEJ1 (XLF), and 53BP1 (Fig. S1F). The presence of key canonical non-homologous end joining (c-NHEJ) genes as top chemo-sensitizer hits validated our experimental approach as this pathway is critical for repairing both drug and ionizing radiation (IR) induced DSBs and provides evidence for a functional c-NHEJ pathway in ES cells. The list of top chemosensitizer genes included Aurora Kinase A (AURKA), for which inhibitors are under clinical development^25^, and the E3 Ubiquitin Ligase RNF8, both of which could be targeted in combinatorial therapeutic approaches with doxorubicin-based treatment regimens. The final category of screen hits were genes whose loss promoted survival under high doses of doxorubicin (LD97), mirroring the residual disease state in ES patients (Fig. S1G). SLFN11 is a notable hit as loss of SLFN11 has been shown to promote chemotherapy resistance in multiple cancer subtypes including ES^26,27^. The complete screen results are provided in Supplementary Table 1. Given the paradoxical finding of HR factor dependence in ES, we chose to focus on the regulation of DSB repair in ES and whether ES tumors are properly classified as BRCA-like cancers.

### ES patient tumors do not display the genomic scars of HR deficiency.

**Figure 2:**
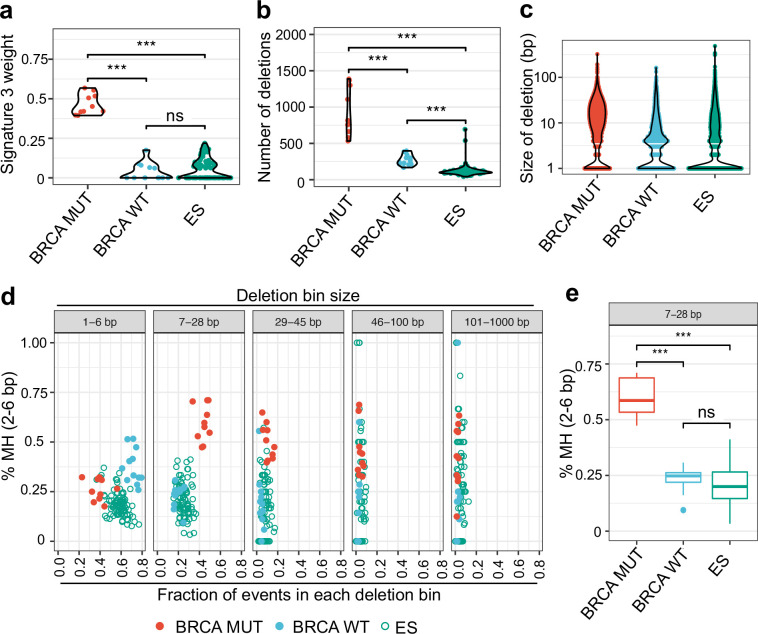
ES patient tumors do not display the genomic scars of HR deficiency. A) Weight of mutational signature 3 shown for BRCA mutant, wildtype and ES tumor samples. B) Total number of unique deletions per sample. C) Size of MuTect deletions compared across tumor groups. D) For all samples, deletions were first subdivided into specific base pair (bp) length bins, then the fraction of deletions with 2–6bp microhomology (MH) was plotted (Y-axis) vs. the fraction of deletions present in this bin (X-axis). E) Breakout of the 7–28bp deletion length bin showing the distribution of deletions with microhomology (2–6bp). For all panels, *** denotes p< 0.001, ns denotes not significant by Mann-Whitney test.

We next directly examined genomic DNA from 99 ES patient tumor samples^28^ for evidence of functional HR deficiency. This is a validated strategy in BRCA1/2 mutant and BRCA-like tumors wherein defective HR repair results in specific genomic scars that result from increased utilization of compensatory pathways such as alternative end-joining (alt-EJ) and single-strand annealing (SSA)^29,30^. We developed a custom bioinformatics pipeline to analyze the genomic landscape of ES tumor genomes, using BRCA1/2 mutant and wildtype breast cancer genomes to validate our algorithms. At least thirty mutational signatures have been identified in human cancers with Signature 3 being the most highly associated with BRCA1/2 mutant cancers^29^. Our analysis confirmed high levels of Signature 3 in BRCA mutant as compared to BRCA wildtype tumors but did not show increased levels of Signature 3 in ES tumors (Figs. 2A and S2A). In addition to increased Signature 3, BRCA1/2 mutant tumors displayed an increase in the number and size of deletions compared to BRCA wildtype tumors (Figs. 2B, C and S2B–D) consistent with previous reports^29^. ES tumors are clearly distinct from BRCA1/2 mutant tumors displaying few deletions per genome and a size distribution skewed further towards small deletions than even BRCA wildtype tumor samples (Figs. 2B, C and S2B–D).

We further examined the sequences flanking deletion sites for short stretches of overlapping microhomology (MH). Defective HR repair in BRCA mutant or BRCA-like tumors results in increased usage of the error prone DSB repair pathway alt-EJ that employs MH for initial annealing of resected DSB ends^31^. We utilized BRCA mutant/wildtype samples to computationally define deletion size bins and identified an increase in the proportion of intermediate size deletions (7–28 base pairs (bp), 29–45 bp) in BRCA mutant samples as reported previously (Figs. S2E, F). We then verified a significant increase in breakpoint MH at intermediate size (7–28bp and 29–45bp) deletions in BRCA mutant tumors compared to wildtype and no increase in MH at small deletions (1–6bp) where c-NHEJ predominates (Figs. 2D, E, and S2E–G), both consistent with published findings^29^. ES samples clustered with BRCA wildtype tumors and display a trend toward even less MH-mediated DSB repair than BRCA wildtype samples (Figs. 2D, E, and S2G). Our findings in ES were independent of the recurrent STAG2 and p53 mutations that occur in a subset of these cancers (Figs. S2H, I). Taken together, these results demonstrate that ES patient tumors do not display the genomic signatures of BRCA1/2 mutant cancers and lack the footprint of isolated HR deficiency.

### EWS-FLI1 impairs resection-dependent DSB repair.

**Figure 3:**
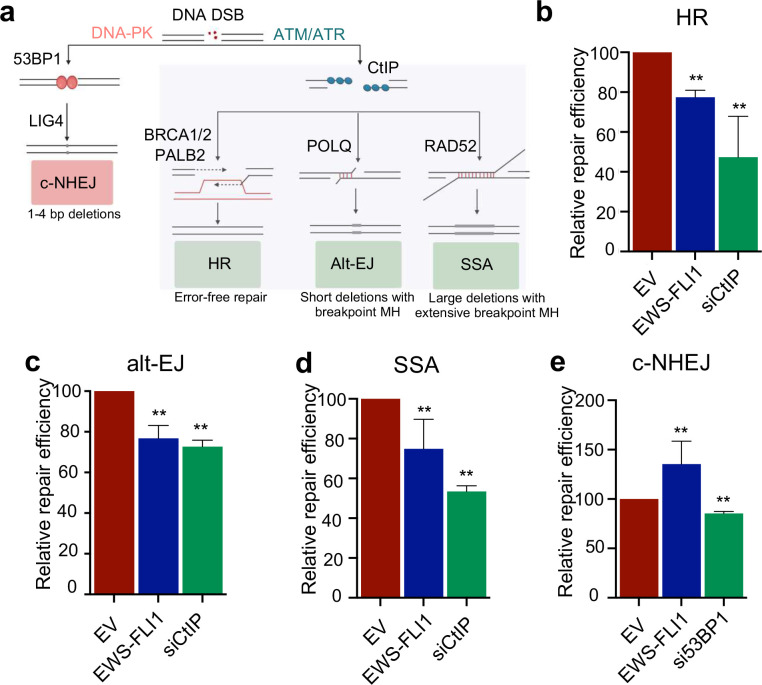
EWS-FLI1 impairs resection-dependent DSB repair. A) Schematic of DSB repair. DSBs either undergo direct repair via c-NHEJ or bidirectional end-resection to create an intermediate for HR, alt-EJ, or SSA. B-E) Relative repair efficiency as measured by GFP positivity in pathway-specific DSB repair reporters for HR (DR-GFP), alt-EJ (EJ2), SSA, and c-NHEJ (EJ5) upon expression of empty vector (EV), EWS-FLI1, or siRNA gene knockdown for 72 hours. For all panels, error bars represent ± SEM, ** denotes p < 0.01 by one-way ANOVA with post hoc Tukey’s HSD test. n=4 for all panels.

The absence of HR deficient genomic scars in ES tumors and paradoxical requirement of HR factors for ES cell survival prompted us to systematically re-examine how EWS-FLI1 impacts DSB repair pathway utilization (Fig. 3A). We posited that previous reports of defective HR in ES might alternatively be explained by a more general upstream defect in DSB repair. We utilized a set of well-established DSB repair reporter cell lines wherein expression of the I-SceI endonuclease induces a DSB within an interrupted GFP reporter cassette, such that utilization of a particular DSB repair pathway restores a GFP coding sequence enabling a quantitative readout of individual repair pathway efficiency^32^. Nucleofection of EWS-FLI1 into each reporter cell line was performed using a BFP-expressing, dual promoter plasmid, followed by expression of mCherry-I-SceI after 24 hours to induce a single DSB (Figs. S3A, B). The use of fluorescently tagged plasmids enabled detection of DSB repair specifically in cells that expressed both EWS-FLI1 (or empty vector) and I-SceI (Fig. S3A).

To examine HR repair, we utilized the DR-GFP reporter and observed a reduction in HR upon EWS-FLI1 expression, consistent with previous reports^10^ (Fig. 3B). However, EWS-FLI1 expression also reduced the efficiency of MH-mediated alt-EJ repair (EJ2) and long-stretch MH mediated-SSA repair (Figs. 3C, D and S3C). EWS-FLI1 mediated reductions in HR, alt-EJ, and SSA repair efficiency were intermediate compared to knockdown of the key end-resection factor CtIP that is required for all resection-dependent DSB repair^33^ (Figs. 3C, D and S3C). These data indicate EWS-FLI1 expression does not result in isolated HR defects, but instead compromises all three resection-dependent DSB repair pathways.

We also evaluated c-NHEJ, a fast-acting DSB repair pathway which does not require end-resection, using the EJ5 reporter system. We observed an increase in the usage of c-NHEJ upon EWS-FLI1 expression (Fig. 3E) consistent with our CRISPRi screen findings of an intact c-NHEJ pathway (Fig. S1F) and the increased frequency of small deletions observed in ES patient samples (Fig. 2C). We verified that these repair phenotypes were not the result of EWS-FLI altering cell cycle profiles or causing a cell cycle arrest (Figs. S3D, E). To assess how EWS-FLI1 regulates the transcription of DDR genes that control resection-dependent DSB repair, we analyzed published RNA-sequencing data in ES cells before and after EWS-FLI1 knockdown^34^ (Fig. S3F). Interestingly, we found that EWS-FLI1 either increased or had minimal effect on the expression of key genes involved in resection-dependent DSB repair (MRE11, CtIP, BRCA1/2, PALB2, RAD52, POLQ), and had no effect on expression of many c-NHEJ genes (LIG4, NHEJ1). These data suggest that EWS-FLI’s effect on DSB repair pathway utilization is not the result of transcriptional downregulation of key end-resection and resection-dependent DSB repair genes (e.g. BRCA1) by the fusion oncoprotein. In summary, we demonstrate that EWS-FLI1 does not induce isolated HR deficiency but instead compromises all three resection-dependent DSB repair pathways.

### ATM activation and signaling is defective in Ewing sarcoma.

To explain our finding that EWS-FLI1 impairs multiple branches of resection-dependent DSB repair, we focused on the upstream regulation of the DDR and end-resection in ES cells (Fig. 3A). The DDR is a partially redundant signaling network regulated by three kinases, DNA-PK, ATM, and ATR, each of which control distinct aspects of DDR signal amplification and DSB pathway choice. ATM was a logical candidate since it promotes DNA end-resection and HR^35^, and ATM loss creates a synthetic lethal dependence on HR proteins^36,37^. We therefore tested whether EWS-FLI1 affects the activation and function of these three apical DDR kinases.

**Figure 4:**
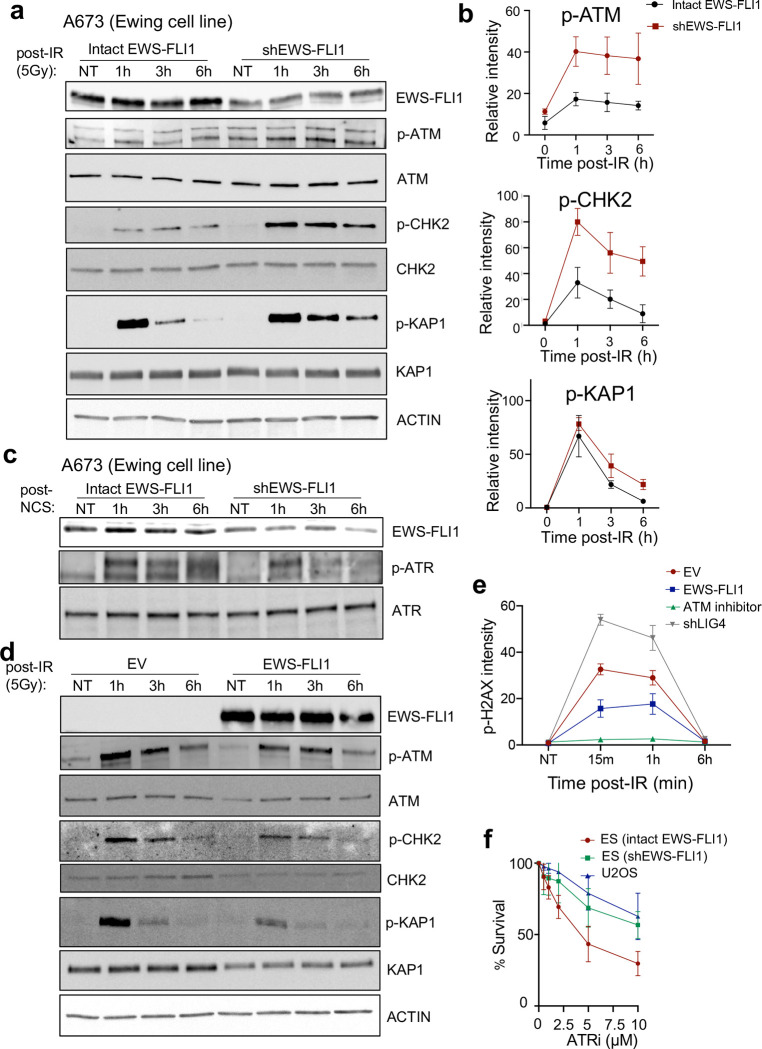
ATM activation and signaling is defective in ES. A, B) Western blotting (A) and quantification (B) after IR (5 Gy) at indicated time points in an ES cell line (A673) with doxycycline-inducible shRNA against EWS-FLI1. h denotes hours. C) Representative Western blot upon 200 ng/ml neocarzinostatin (NCS) treatment for optimal ATR induction at indicated time points in an ES cell line A673 with doxycycline(dox)-inducible shRNA against EWS-FLI1. D) Representative Western blot upon IR (5 Gy) at indicated time points in non-ES cancer cell line (U2OS) upon expression of EV (empty vector) or EWS-FLI1. Quantification of Western blotting in Figures S4B–S4D. E) p-H2AX intensity by flow cytometry in non-ES (U20S) cells post-IR (5 Gy) after expression of EV or EWS-FLI1. Control experiments with ATMi treatment (KU-55933, 1μM) or shRNA against Ligase IV (LIG4), p < 0.05 for all comparisons using one-way ANOVA with post hoc Tukey’s HSD test. F) ATR inhibitor (VE-821) dose-response curves for ES cell line A673 with dox-inducible shRNA against EWS-FLI1 and non-ES cell line (U2OS), p<0.05, paired t-test. For all panels, error bars represent ± SEM for at least 3 replicates for each panel.

Transient knockdown of EWS-FLI1 in ES cells increased both ATM activation (autophosphorylation) and to a greater extent, downstream ATM signaling (phosphorylation of key downstream targets CHK2 and KAP1) in response to ionizing radiation (IR) (Figs. 4A, B). In contrast, activation of the other apical DDR kinases was either unaffected (DNA-PK) or decreased (ATR) by EWS-FLI1 knockdown in ES cells (Figs. 4C and S4A). In the reciprocal experiment, expression of EWS-FLI1 in a non-ES cancer cell line (U2OS) suppressed ATM activation and downstream ATM signaling in response to IR (Figs. 4D and S4B–S4D), without affecting DNA-PK or ATR activation and signaling (Figs. S4E, F). Absolute p-ATM foci number after IR was not affected by EWS-FLI1 (Fig. S4G) and we observed a reduction in pH2AX signal intensity by flow cytometry, but not absolute pH2AX foci number, after IR in EWS-FLI1 expressing cells (Figs. 4E and S4H). These data further support an ATM signaling defect in ES as ATM is the principal DDR kinase responsible for the phosphorylation and amplifying spread of pH2AX surrounding DSBs^35^.

Both ATM and ATR coordinate aspects of resection-dependent DSB repair (Fig. 3A) and ATM mutant tumors display synthetic lethality with ATR inhibitors, reflecting the vital compensatory role of the ATR signaling axis in the absence of ATM^38,39^. We therefore tested if functional ATM deficiency caused by EWS-FLI1 induced a collateral dependence on the ATR signaling axis in ES given this emerging synthetic lethal relationship between the two DDR kinases. Indeed, ES cells displayed increased sensitivity to inhibitors of both ATR and its key downstream target CHK1, and inhibitor sensitivity was reversed by EWS-FLI1 knockdown in both cases (Figs. 4F and S4I). Overexpression of RNAseH1 which degrades R-loops, the major source of oncogene-induced replication stress in ES^10^, did not alter the ATR inhibitor sensitivity of ES cells (Figs. S4J, K) suggesting that the molecular basis of ATR inhibitor response in ES cells may be a consequence of functional ATM deficiency. The collective findings establish an EWS-FLI1-dependent specific impairment of ATM function in ES and resultant synthetic lethality with the compensatory ATR signaling axis.

### Loss of native EWS phenocopies the DNA repair defects caused by EWS-FLI1.

How does EWS-FLI1 regulate DNA repair to create ATM defects? We hypothesized that previously reported interactions between EWS-FLI1 and native FET proteins (e.g. EWS) may disrupt the putative function of native FET family members in early DSB repair compartmentalization^40,41^, thereby impacting ATM activation and downstream signaling. Consistent with prior studies^41^, EWS-FLI1 and another native FET family member (FUS) coprecipitated with FLAG-tagged EWS (Fig. S5A). The strength of these interactions was unchanged in the setting of the DNA DSB-inducing agent neocarzinostatin (NCS) indicating a high affinity interaction not disrupted by the DDR. Given that native EWS accumulates at laser-induced DSB stripes within seconds^15^, we tested whether the aberrant interaction between EWS and EWS-FLI1 in ES impacts the kinetics of native EWS recruitment to DNA DSBs.

**Figure 5:**
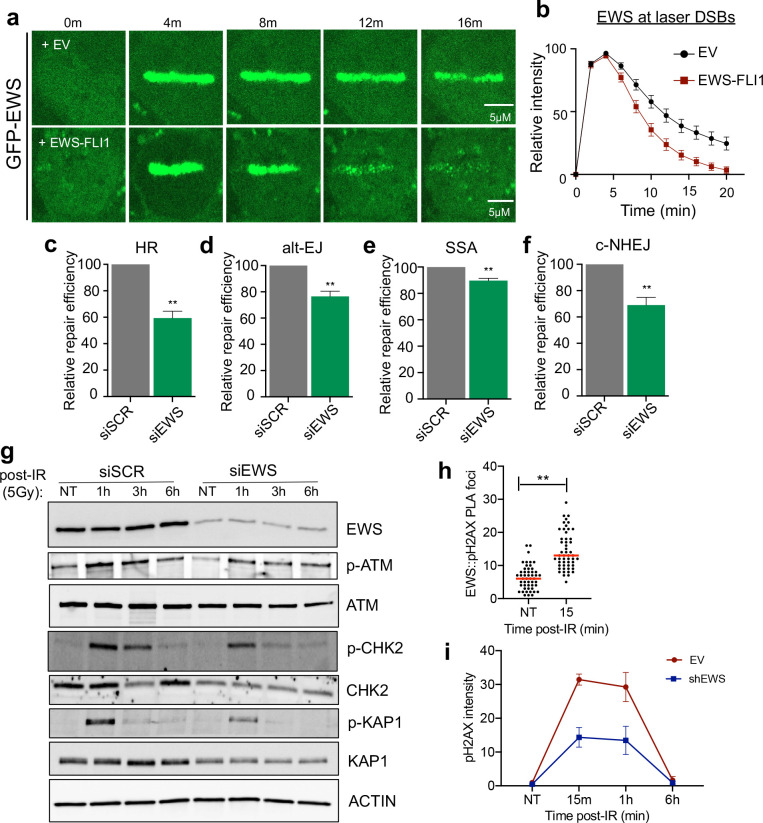
Loss of native EWS phenocopies the DNA repair defects caused by EWS-FLI1. A, B) Representative images and quantification of laser micro-irradiation in GFP-EWS expressing non-ES cells (U2OS) +/− EWS-FLI1 expression. n = 40 cells. C-F) Relative repair efficiency in pathway-specific DSB repair reporters in U2OS cells. GFP positivity for HR (DR-GFP), alt-EJ (EJ2), SSA, and c-NHEJ (EJ5) upon siRNA knockdown of native EWS or siScramble (siSCR) control assessed at 72 hours. G) Representative Western blot upon IR (5Gy) at indicated time points in a non-ES cell line(U2OS) after 72 hours siRNA treatment (siEWS or siSCR). Quantification of Western blotting shown in Figure S5C–S5E. H) Proximity ligation assay (PLA) between native EWS and p-H2AX in U2OS cells upon IR (10Gy) at indicated time points. I) p-H2AX intensity measured by flow cytometry after 5 Gy IR in U20S cells infected with shRNA against native EWS or empty vector (EV) control. p<0.05 by paired t-test. For all panels, error bars represent ± SEM, ** denotes p < 0.01 by paired t-test with at least 3 replicates for each panel.

The initial recruitment of EWS to laser micro-irradiated DSBs was unaffected by EWS-FLI1. Instead, EWS-FLI1 expression resulted in the premature and rapid clearance of EWS from DSB stripes (Figs. 5A, B). To determine whether loss of native EWS function at DNA DSBs may contribute to EWS-FLI1-dependent DNA repair defects, we first characterized the role of native EWS in DSB repair using the pathway-specific DSB repair reporters. Analogous to EWS-FLI1 expression, native EWS knockdown decreased all three resection-dependent DSB repair pathways, HR, alt-EJ and SSA (Figs. 5C–E and S5B). Interestingly, c-NHEJ efficiency was also reduced upon EWS knockdown (Fig. 5F), highlighting differences between native EWS loss and EWS-FLI1 overexpression that may relate to the rapid kinetics of c-NHEJ repair and preserved initial recruitment of native EWS despite EWS-FLI1 expression.

We further assessed the role of native EWS in activation of the upstream DDR kinases ATM, DNA-PK, and ATR. Analogous to EWS-FLI1 expression, native EWS knockdown reduced activation of ATM itself (autophosphorylation) and to a greater extent, functional ATM signaling (phosphorylation of CHK2 and KAP1) upon IR, again with either no effect (DNA-PK) or increased (ATR) activation of the other two DDR kinases (Figs. 5G and S5C–G). We utilized a proximity ligation assay (PLA) to establish an IR-dependent interaction between pH2AX and EWS (Figs. 5H and S5H), further supporting a role for native EWS in coordinating early DSB repair responses. Finally, knockdown of native EWS also decreased IR-induced pH2AX signal intensity by flow cytometry, but not pH2AX foci number, analogous to EWS-FLI1 overexpression (Fig. 5I). These data define the function of native FET family member EWS as a specific regulator of ATM activation and signaling. In total, we demonstrate that EWS-FLI1 causes premature loss of native EWS from DSBs and functional ATM defects that are phenocopied by native EWS knockdown.

### EWS-FLI1 and other FET fusion oncoproteins are recruited to DNA double-strand breaks.

**Figure 6:**
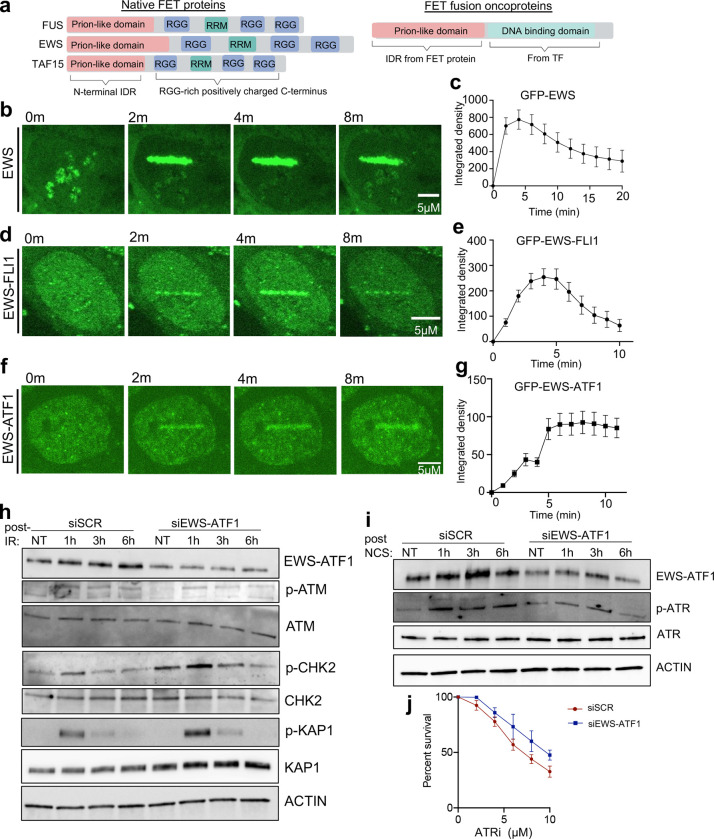
FET fusion oncoproteins are recruited to DNA DSBs. A) Structure schematic of FET proteins with N-terminal intrinsically disordered regions (IDR) and C-terminal Arginine-glycine-glycine (RGG) repeats, not present in FET fusion oncoproteins like EWS-FLI1. B-G) Representative images and quantification of protein accumulation at laser-induced DSBs in U2OS cells expressing either GFP-EWS (B, C), GFP-EWS-FLI1 (D, E), or GFP-EWS-ATF1 (F, G). Quantification of at least 30 cells in 3 replicates. H, I) Representative Western blot upon 5 Gy IR (H) or 200 ng/ml NCS treatment (I) at indicated time points in CCS cell line SU-CCS-1 upon 72 hour siRNA treatment against EWS-ATF1 or Scramble (siSCR) control. Quantification in Fig. S6D–F. I) ATR inhibitor (VE-821) dose-response curves for CCS cell line SU-CCS-1 upon siRNA against EWS-ATF1 or siSCR, p<0.05 by paired t-test, n-3. For all panels, error bars represent ± SEM, at least 3 replicates for each panel.

To determine a molecular basis for the ATM defects seen in ES, we asked whether the interaction between native EWS and EWS-FLI1 (Fig. S5A) might promote aberrant localization of the fusion oncoprotein to DNA DSBs. Indeed, we discovered that EWS-FLI1, like native EWS, is recruited to laser-induced DSBs (Figs. 6B–E). The kinetics of EWS-FLI1 recruitment were delayed compared to native EWS, which accumulates within seconds due to C-terminal, positively charged RGG domains not present in EWS-FLI1 (Figs. 6A–E). Importantly, recruitment to DSBs represents an unanticipated neomorphic property of the fusion protein contributed by the N-terminal IDR of EWS. Control experiments with the full-length FLI1 protein showed no accumulation at laser-induced DSBs (Fig. S6A). Consistent with the N-terminus of EWS mediating DSB recruitment, mutation of the DNA binding domain of FLI1 (R2L2 mutant of EWS-FLI1^42^) had no effect on the DSB localization of EWS-FLI1 (Fig. S6B). Furthermore, knockdown of native EWS reduced accumulation of EWS-FLI1 at laser-induced DSB stripes, indicating that the localization of EWS-FLI1 to DSBs depends, at least in part, on native EWS (Fig. S6C).

Lastly, we tested the generality of the concept that N-terminal IDRs, as a shared structural feature of FET fusion oncoproteins (Fig. 6A), could promote aberrant DSB recruitment in other tumors within this class. EWS-ATF1 is the sole oncogenic driver of clear cell sarcoma (CCS) and contains the identical N-terminal IDR sequence as EWS-FLI1. The EWS-ATF1 fusion oncoprotein localized to laser-induced DSBs with delayed recruitment kinetics similar to EWS-FLI1 though with differences in departure timing (Figs. 6F, G). Analogous to our findings in ES, transient knockdown of EWS-ATF1 in a patient-derived CCS cell line increased downstream ATM signaling (phosphorylation of ATM targets CHK2 and KAP1) in response to ionizing radiation (IR), with either no effect (DNA-PK) or compensatory decrease (ATR) in activation of the other upstream DDR kinases (Figs. 6H, I and S6D–S6G). These data also reveal differences between FET fusion oncoproteins; only EWS-FLI1 affected the initial activation of ATM (autophosphorylation), while both EWS-ATF1 and EWS-FLI1 caused functional ATM defects in terms of downstream signaling. Despite these differences, the consequence of functional ATM deficiency in CCS was similarly increased reliance on compensatory ATR signaling, as CCS cells displayed EWS-ATF1-dependent synthetic lethality with ATR inhibition (Fig. 6J). Taken together, our results support a model in which EWS fusion oncoproteins are aberrantly recruited to DSB repair sites and induce specific defects in ATM function.

## Discussion

Here we detail a new mechanism for how oncogenes can create tumor-specific vulnerabilities in DNA damage repair networks: aberrant recruitment of an oncogenic fusion protein to sites of DNA damage causing disruption of normal DSB repair biology. We identify functional ATM deficiency as the principal DDR defect in ES and demonstrate oncogene-dependent synthetic lethality with ATR inhibition as a collateral dependency. These data provide an initial example of how DDR-directed therapies could be utilized in the broader set of cancers without genetic DDR alterations through improved understanding of how oncogenes interact with DNA damage networks.

The nature of the DNA repair defect in ES has been the subject of much debate based on the strong clinical and laboratory-based data demonstrating chemo- and radio-sensitivity^6–10^. Our discovery that ES tumors are not “BRCA-like”, but instead functionally ATM deficient, may help explain the lack of clinical responses to PARP inhibitor monotherapy in ES patients^11,43^ (unlike HR deficient BRCA mutant and BRCA-like cancers^12,13^). ES cells are dependent on key HR genes for survival and direct analysis of ES patient tumors revealed none of the genomic hallmarks of HR loss. Instead of isolated HR deficiency, we show that EWS-FLI1 creates a broader defect in resection-dependent DSB repair and a specific impairment of upstream ATM activation and signaling. ES cells display increased reliance on a compensatory DDR kinase, ATR, and EWSFLI1-dependent synthetic lethality with ATR and CHK1 inhibitors. These data nominate functional ATM deficiency as the principal DNA repair defect in ES.

**Figure 7:**
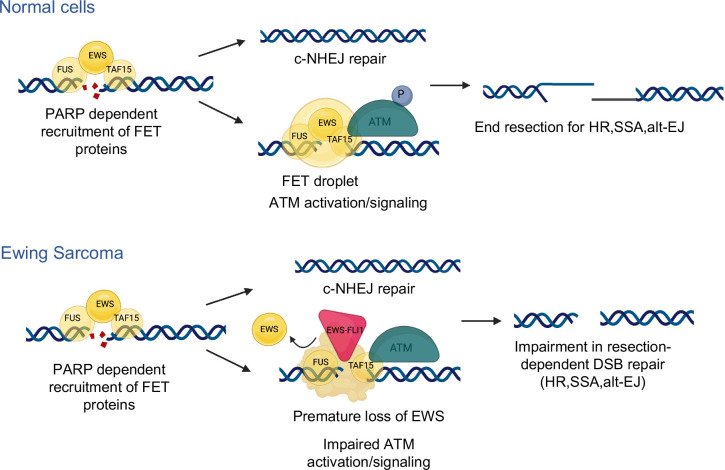
Model of native FET proteins and EWS-FLI1 in DSB repair. Schematic of FET proteins coordinating early DSB repair responses including rapid c-NHEJ repair and slower ATM activation and resection-dependent DSB repair. In ES, EWS-FLI causes premature loss of native EWS resulting in impaired ATM activation/signaling and resection-dependent DSB repair.

We propose a model wherein the rapid recruitment of native FET proteins (e.g. EWS) are important for c-NHEJ, consistent with our findings upon native EWS knockdown and published reports on FUS’s role in canonical repair^44^ (Fig. 7). The FET proteins then coordinate local compartmentalization of DSB repair factors leading to ATM chromatin recruitment and activation, amplification of the ATM signaling cascade and phosphorylation of key substrates (e.g. CHK2), and slower resection-dependent repair. Our work raises important new questions as to how native FET proteins interact with DDR scaffolding proteins and how IDR-mediated phase separation might enable DSB compartmentalization and subsequent repair. In ES, we show that the EWS-FLI1 fusion oncoprotein is recruited to DNA DSBs via homotypic IDR interactions with native EWS and results in the premature departure of native EWS from DSB sites. Our finding that loss of EWS largely phenocopies the DNA repair defects caused by EWS-FLI1 implicates the interaction between shared N-terminal IDRs of EWS and EWS-FLI1 as central to the DNA repair defects in ES. The extent to which the hemizygosity of native EWS in ES tumors contributes to DNA repair phenotypes and how EWS-FLI1 disrupts the local compartmentalization and phase separation of native FET proteins at DSBs are important questions raised by our findings.

More generally, the shared structural organization across the class of FET fusion oncoproteins (i.e. retention of N-terminal IDRs that mediate homotypic and heterotypic FET protein interactions and loss of C-terminal RGG domains that promote rapid DSB recruitment, Fig. 6A) raises the intriguing hypothesis that FET fusion-driven cancers harbor a common set of DNA repair defects. Indeed, our finding that the clear cell sarcoma fusion oncoprotein EWS-ATF1 localizes to DNA DSBs and induces specific functional ATM defects supports a more general role for FET fusion oncoproteins in regulating and disrupting DNA DSB repair. Analogous to our findings in ES, CCS cells also display FET fusion oncogene-dependent synthetic lethality with ATR inhibitors. These data support expanded testing of ATR inhibitors across the class of FET rearranged cancers as a therapeutic strategy to specifically target functional ATM deficiency induced by FET fusion oncoproteins.

Finally, what might be the selective advantage for TF fusion oncoproteins to also induce a DNA repair deficiency? We propose that by partially disabling physiologic DDR signaling, cells with FET rearrangements can tolerate high levels of DDR activation caused by transcription and replication stress induced by the oncoprotein itself and thus overcome an important barrier to cellular transformation. Whether other TF fusion oncoproteins (or oncogenes more generally) interact with and disrupt DDR signaling networks as part of tumorigenesis will be an intriguing topic for future work. In summary, our study provides a new mechanism for how oncogenes interact with DNA repair networks by detailing the aberrant recruitment of an oncogenic fusion protein to DSBs and disruption of native DSB repair biology. This work provides rationale for new targeted therapeutic strategies (e.g. ATM/ATR synthetic lethality) in ES and the broader class of undruggable FET fusion-driven cancers, and a roadmap for how DDR-directed therapeutics can be rationally deployed through an improved understanding of how oncogenes interact with DNA repair networks.

## Methods

### Cell culture.

U2OS and 293T cells were cultured in Dulbecco’s Modified Eagle Medium (DMEM)-High glucose (Cytiva) supplemented with 10% fetal calf serum (FCS) and streptomycin/penicillin (100 μg/ml). A673, TC71, ES8, SU-CCS-1 and HCC364 cells were grown in RPMI-1640 (Gibco) supplemented with 10% FCS and streptomycin/penicillin (100 μg/ml). A673 cells with doxycycline(dox)-inducible shRNA against EWS-FLI1 were a kind gift from Olivier Delattre^28^ . Cell lines were maintained at 37 °Celsius in a humidified incubator with 5% CO2. All cell lines were subjected to STR analysis and mycoplasma testing.

### CRISPR-interference screen.

Ewing sarcoma cell line A673 was transduced with dCas9-KRAB-BFP and then doubly sorted for BFP positive cells. Lentiviral particles were generated from 293T cells transduced with pooled sgRNA libraries (7000 genes, 10 genes per guide) as described previously^21^ and then used to infect A673 dCas9-KRAB cells at a multiplicity of infection of 0.3. Cells were selected in puromycin for 48 hours and an aliquot was frozen for t_0_ analysis. The remainder of cells were seeded in 500 cm^2^ tissue culture plates at equal density (1 × 10^7^ cells/plate) and then treated with vehicle (DMSO) or 12.5 nM/40 nM doxorubicin for 72 hours. After 72 hours, cells were trypsinized, counted and pooled every 3 days, and then replated in media without doxorubicin at the same density to maintain minimum 500× coverage of each sgRNA construct. Cells were viably frozen after 10 days and cell counts were used to determine actual lethal dose (LD) values. 2 biologic replicates were performed, and data combined.

Deep sequencing and data analysis were performed as described^21^. Briefly, genomic DNA was extracted from t_0_ and t_end_ cells using a DNEasy Blood & Tissue kit (Qiagen), digested to enrich for lentiviral integration sites, and sgRNA sequences were amplified by PCR for subsequent sequencing on an Illumina HiSeq. Reads were aligned to the sgRNA library and fold-change from t_0_ to t_end_ in the DMSO, low and high doxorubicin conditions was calculated. A gene-level score was then calculated as the mean of the top 3 scoring sgRNAs targeting a given transcript.

### CRISPRi growth assays.

dCas9-KRAB expressing cells were infected with gRNAs (sequences below) and then selected with puromycin for 48 hours. 50,000 cells were then re-plated in a 6 well plate and cell counts were obtained by trypsinization and counting at day 5. For experiments in A673 cells with dox-inducible shRNA against EWS-FLI1, 1μg/ml Dox (or vehicle) was added upon replating the puromycin selected, gRNA-expressing cells. gRNA sequences are as follows:

sgBRCA1-1: GCGTAAGGCGTTGTGAACCCT

sgBRCA1-2: GCTCGCTGAGACTTCCTGGAC

sgPALB2-1: GCGCACTGAGGGTGCGATCC

sgPALB2-2: GATTTAATTGGCCGGAGTTT

sgControl-1: GGCTCGGTCCCGCGTCGTCG

sgControl-2: GAACGACTAGTTAGGCGTGTA

### Computational analysis of genomic data.

Whole genome sequencing data from known BRCA mutant breast cancer patient samples and Ewing sarcoma samples were obtained from EGA (EGA datasets: EGAD00001001322 and EGAD00001001051, respectively). FASTQ files were aligned to the GRCh38 (patch 5) human genome reference using BWA MEM^45^, with pre- and post-alignment filtering and processing using NGSUtils^46^ and GATK^47^. Insertions and deletions (indel) were called using MuTect 2 for short indels^48^ and Delly for larger indels^49^. For each indel, the amount of microhomology at the flanking ends was calculated using the custom software “mhscan”. Briefly, for each indel, mhscan will determine the number of bases of homology between the insert or deletion and the flanking sequence. All indels with between 2–6 bp of homology were classified as microhomology (MH) positive. Mutational signatures were calculated using the somatic single-nucleotide variants for each sample. COSMIC v2^50,51^ signatures were calculated using deconstructSigs^52^.

### Determining deletion length bins.

The binning ranges were determined by calculating the ability for the number of deletions of each length (1–100 bp) to accurately separate the BRCA wild type (wt) / mutant (mut) samples into two groups. The deletions were classified as either MH-positive or MH-negative and the total number of deletions for each length was calculated for BRCA wt and BRCA mut samples (data were aggregated for all samples in the group). For each deletion length, a Fisher exact test was used to calculate the statistical significance of whether the MH-positive to MH-negative ratio separated the BRCA wt/mut groups (Fig. S2F). The distribution of p-values was used to determine appropriate bin sizes for downstream analysis. Deletions were binned into ranges of 1–6 bp, 7–28 bp, 29–45 bp, 46–100 bp, and 101–1000 bp.

### DSB Repair reporter assays.

HR, Alt-EJ, SSA and c-NHEJ efficiencies were measured using previously established DR-GFP, EJ2-GFP, SA-GFP and EJ5-GFP reporter systems respectively^32^. Briefly, dual promoter plasmids co-expressing mTagBFP and EWS-FLI1 (or empty vector, EV) were generated by replacing the puromycin cassette with mTagBFP in the plasmid pCDH-CMV-EWS-FLI1 (Addgene #102813). Co-expression plasmids were delivered by nucleofection into U2OS DSB reporter cell lines using the SE Cell Line 4D-Nucleofector XL Kit (Lonza Biosciences). To induce a DSB, the I-SceI-P2A-mCherry plasmid which we generated by replacing AmCyan with I-SceI in the bicistronic plasmid Amcyan-P2A-mCherry (Addgene #45350) was delivered by nucleofection after 24 hours. Flow cytometry was performed 72 hours after I-SceI introduction. DSB repair efficiency was calculated by determining the percentage of GFP positive cells within the BFP and mCherry double-positive population (see Fig. S3A). For siRNA experiments, siRNA (5nM) was delivered by transfection using Dharmafect transfection reagent (Dharmacon) 72 hours prior to I-SceI introduction.

### Western blotting.

Cells were washed with 1X PBS and scraped in RIPA buffer (25 mM Tris HCl (pH 7.6), 150 mM NaCl, 1% NP-40, 1% sodium deoxycholate, 0.1% SDS), supplemented with 1X HALT protease inhibitor cocktail and 1X HALT phosphatase inhibitor cocktail. Cells were lysed using a syringe and cellular debris were separated by centrifugation. Lysates were quantified using Bradford’s reagent and 15μg lysate was loaded onto SDS-PAGE gels followed by blotting of separated proteins. Blots were blocked with 5% BSA in TBS-T for 30 minutes at room temperature followed by incubation with primary antibody overnight at 4°C. After 1 hour incubation with appropriate HRP-conjugated secondary antibody, signal was detected using ECL Prime reagent (Amersham, Cytiva) on an ImageQuant LAS 4000. Quantifications were performed using Fiji and all protein levels were normalized to loading control Actin.

The list of antibodies utilized in this study includes: FLI1 (Abcam, ab84078, 1:200), CtIP (Cell Signaling Technologies (CST), D76F7, 1:000), 53BP1(Abcam, ab17593, 1:1000), pATM (CST, 4526S,1:1000), ATM (CST, 2873S, 1:1000), pCHK2-T68 (CST, 2661S, 1:1000), CHK2 (CST, 2662S, 1:1000), pKAP1 (Novus Biologicals, A700–013, 1:1000), KAP1 (Novus Biologicals, A700–014, 1:1000), pDNA-PK (CST, 68716S, 1:1000), DNA-PK (CST, 4602T, 1:1000), pATR (Genetex, GTX128145, 1:1000), ATR (Novus Biologicals, A300–138A, 1:1000), GFP (CST, 2956S, 1:1000), EWSR1 (Genetex, GTX114069, 1:1000), beta-Actin (Sigma, A2228, 1:2000), anti-Flag (Sigma, F3165, 1:2000), ATF1 (Novus Biologicals, A303–036A, 1:1000), FUS (Novus Biologicals, A300–302A-1:1000), RNaseH1 (Genetex, GTX117624, 1:1000).

### DDR signaling experiments.

For experiments in U20S cells, the respective fusion proteins (EWS-FLI1-GFP, EWS-ATF1-GFP, or EV-GFP) were introduced via nucleofection with homemade buffers (Solution 1: 2g ATP-disodium salt, 1.2g MgCl_2_.6H_2_0 in 10ml water; Solution 2: 6g KH_2_PO_4_, 0.6g NaHCO_3_, 0.2g Glucose in 500ml water, pH 7.4. Solutions were stored at −20°C and 80μl of Solution1 was mixed with 4ml of solution 2 at the time of nucleofection). GFP positive cells were selected by FACS. 24 hours after sorting, cells were subjected to 5Gy IR using a Cs-137 irradiator. Lysates were collected at 1, 3, and 6 hours post-IR and subjected to Western blotting. For experiments in A673 cells with inducible shRNA against EWS-FLI1, knockdown was induced by addition of 1μg/ml Dox for 72 hours followed by IR treatment. For experiments in SU-CCS-1 cells, siRNA against EWS-ATF1 was delivered by nucleofection 72 hours prior to IR. For improved induction of ATR/CHK1 signaling, cells were treated with neocarzinostatin (200ng/mL) for 30 minutes and then collected at 1, 3, and 6 hours after removing the drug.

### siRNA/shRNA sequences.

siEWS: Mix of two siRNAs targeting the C-terminus of EWSR1: GGAACAGAUGGGAGGAAGA and AGGAAAGCCCAAAGGCGAU

siCTIP: On-TARGETplus SMARTpool siRNA (Dharmacon, L-011376–00-0005)

si53BP1: On-TARGETplus SMARTpool siRNA (Dharmacon, L-003548–00-0005)

siEWS-ATF1: GCGGUGGAAUGGGAAAAAUUU

shRNA sequence against native EWS: TGCATTGACTACCAGATTTAT

shRNA sequence against LIG4: TATGTCAGTGGACTAATGGAT

### Cell survival assays.

350,000 cells were seeded in 6-well plates, 24 hours prior to drug treatment. Cells were treated with varying doses of ATR kinase inhibitor VE-821 (Selleckchem) or CHK1 inhibitor LY2603618 (Selleckchem) for 3 days at the end of which cells were counted to determine fractional cell survival. For dox-inducible EWS-FLI1 knockdown in A673 cells, shRNA was induced by treatment with 1μg/ml dox for 72 hours prior to ATR or CHK1 inhibitor treatment. For RNAseH1 experiments, A673 cells were infected with pLV-EF1a-RNAseH1-IRES-Blast lentivirus (derived from Addgene plasmid #85133) or empty vector and selected for stably infected cells.

### pH2AX flow cytometry.

EWS-FLI1 (or EV) expressing U20S cells were obtained by nucleofection as above. U20S cells with shRNA against LIG4 or native EWS were obtained through puromycin selection with pLKO.1 shRNA expressing vectors with sequences as above. Cells were treated with 10Gy IR and harvested at 15 minutes and 1, 3, 6 hours post-IR. After washing with 1ml ice cold PBS, cells were resuspended in 300μl 1X PBS and 700μl of 100% ice-cold ethanol dropwise while gently vortexing the tubes and then incubated overnight at −20°C. The next day, cells were washed with 1x PBS and incubated for 15min at room temperature (RT) in 1.5 mL wash buffer (1% BSA containing 0.25% Triton X-100 in PBS). After centrifugation, cells were incubated in 200 μL of α-phospho H2AX FITC conjugate (JBW301, Millipore,1:500 in wash buffer) for 2 hours at RT in the dark. Cells were then resuspended in 300–500 μl PI solution for 60 min at RT in the dark prior to FACS analysis.

### Laser micro-irradiation.

Laser micro-irradiation was performed as previously described^16^. Briefly, U20S cells expressing EWS-GFP, EWS-FLI1-GFP, or EWS-ATF1-GFP were seeded in 8-well Lab Tek II Chamber Slides (Thermo Fisher Scientific) for 24 hours. Cells were treated with 1μg/ml Hoechst 33342 (Thermo Fisher Scientific) for 30 minutes prior to micro-irradiation. Live cell microscopy was performed using Nikon Ti microscope with a CSU-W1 spinning disk confocal using a 100X/1.4 Plan Apo VC objective. To induce DNA damage, 5 pixel wide stripes were drawn in every cell nucleus to label the region of interest (ROI) and irradiated with a 405nm diode laser (40mW). Images were acquired pre-irradiation and at 1 minute intervals post-laser damage for 15 minutes. To plot recruitment kinetics, the pre-irradiation fluorescence intensity was subtracted from the intensity of the ROI for every nucleus.

### Proximity ligation assay.

U2OS cells were seeded on autoclaved coverslips and subjected to 10Gy IR. 15 minutes post-IR, cells were washed twice with 1X PBS and fixed with 4% formaldehyde for 15 minutes at room temperature. Fixed cells were incubated with 0.5% Triton X-100 for 10 minutes. Cells were then washed twice with 1X PBS and blocked in 5% BSA/1X PBS solution for 1 hour at room temperature. Coverslips were then incubated overnight at 4 °C with EWS (Origene, 1:200) and pH2AX (CST, 1:500) antibodies. The following day proximity ligation assay (PLA) was performed using Duolink PLA technology (Sigma-Aldrich) according to the manufacturer’s instructions. Images were acquired using Nikon Ti microscope with a CSU-W1 spinning disk confocal using a 100X/1.4 Plan Apo VC objective (Center for Advanced Light Microscopy, UCSF).

### EWS Co-Immunoprecipitation (co-IP).

293T cells were transfected with Flag-EWS and EWS-FLI1 (or empty vector) for 48 hours. Cells were then treated with NCS (200ng/mL) for 30 minutes and cells were collected at 15 minutes, 30 minutes and 1 hour post NCS treatment. Nuclear co-IP was performed using the Nuclear Co-IP kit (Active Motif) according to the manufacturer’s instructions.

### Cell cycle analysis.

U2OS cells were nucleofected with the dual promoter pCDH-EWS-FLI1-EF1a-mtagBFP (or empty vector) and analyzed at 48 hours. Cells were fixed using 4% formaldehyde for 15 minutes at room temperature. After washing with 1% BSA in PBS, cells were resuspended in 500μl of FxCycle^™^ PI/RNAse Solution (ThermoFisher Scientific) for 30 minutes at room temperature. Cell cycle profiles were also analyzed using Click-iT EdU Alexa Fluor 488 Flow Cytometry Assay Kit (Invitrogen). Cells were pulse labelled with 10μM EdU for 30 minutes and processed according to the manufacturer’s instructions. Flow cytometry was used to analyze the cell cycle profile for BFP positive, EWS-FLI1 (or empty vector) expressing cells.

### Statistics.

Data from at least three independent experiments were used to calculate P values which were determined with Student’s t tests and ANOVA using GraphPad software.

## Supplementary Material

Supplement 1
