# Supplementary material for: FET fusion oncoproteins disrupt physiologic DNA repair networks and induce ATR synthetic lethality in cancer
Source: Res Sq. 2023 May 29:rs.3.rs-2869150. Preprint. [Version 1] doi: 10.21203/rs.3.rs-2869150/v1 (PMC10312925; doi:10.21203/rs.3.rs-2869150/v1)
Supplement: Supplement 1 [file NIHPPrs2869150v1-supplement-1.pdf]

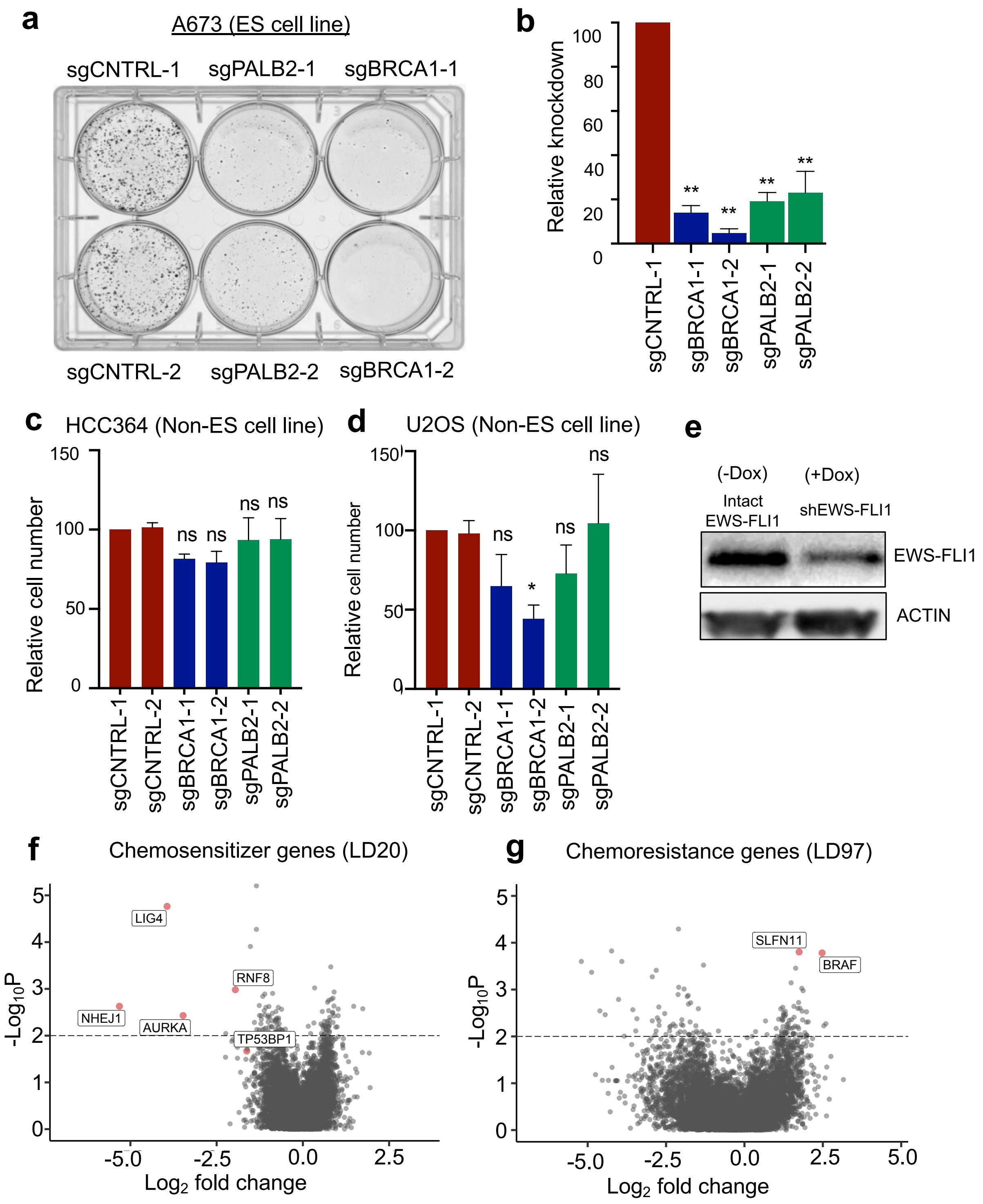

Supplemental Figure 1: ES cells are dependent on HR factors for survival.
A) Representative crystal violet staining of 72 hour growth assay in dCas9 expressing A673 ES cells upon introduction of 2 independent sgRNAs against BRCA1, PALB2, or control.
B) Relative knockdown of each sgRNA by qPCR, n = 3.

C, D) Growth assays in dCas9 expressing cancer cell lines upon introduction of 2 independent sgRNAs against BRCA1, PALB2, or control, n = 4, 4.
E) Western blot of EWS-FLI1 levels in A673 with doxycycline-inducible shRNA against EWS-FLI1.
F, G) Volcano plot of sgRNA $\log_2$fold change relative to DMSO control (X-axis) vs -log10(p value). Full screen results provided in Supplementary Table 1.
For all panels, * denotes $p< 0.05$, ** denotes $p < 0.01$, ns denote not significant by one-way ANOVA with post hoc Tukey's HSD test.

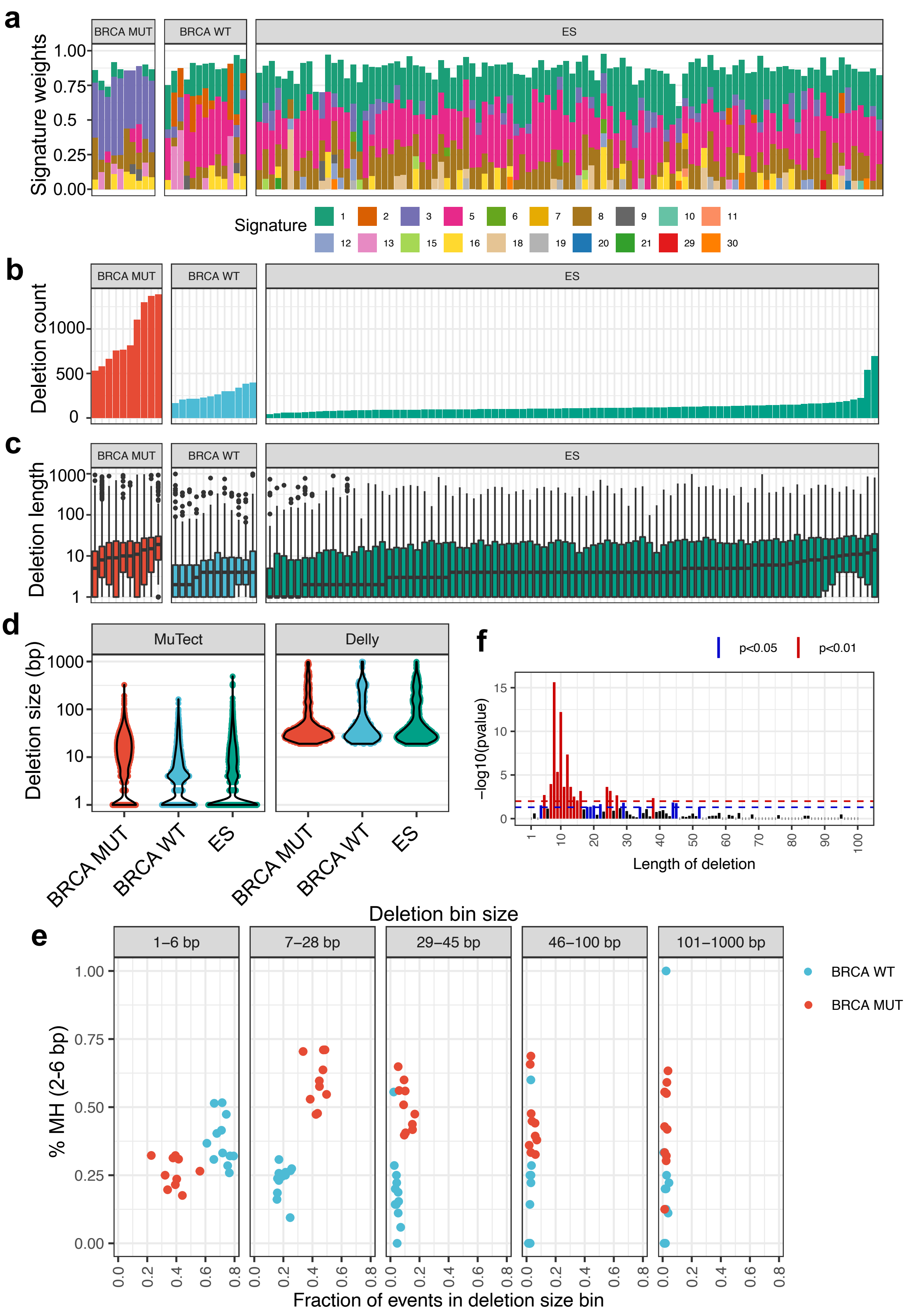

Supplemental Figure 2 continues on next page

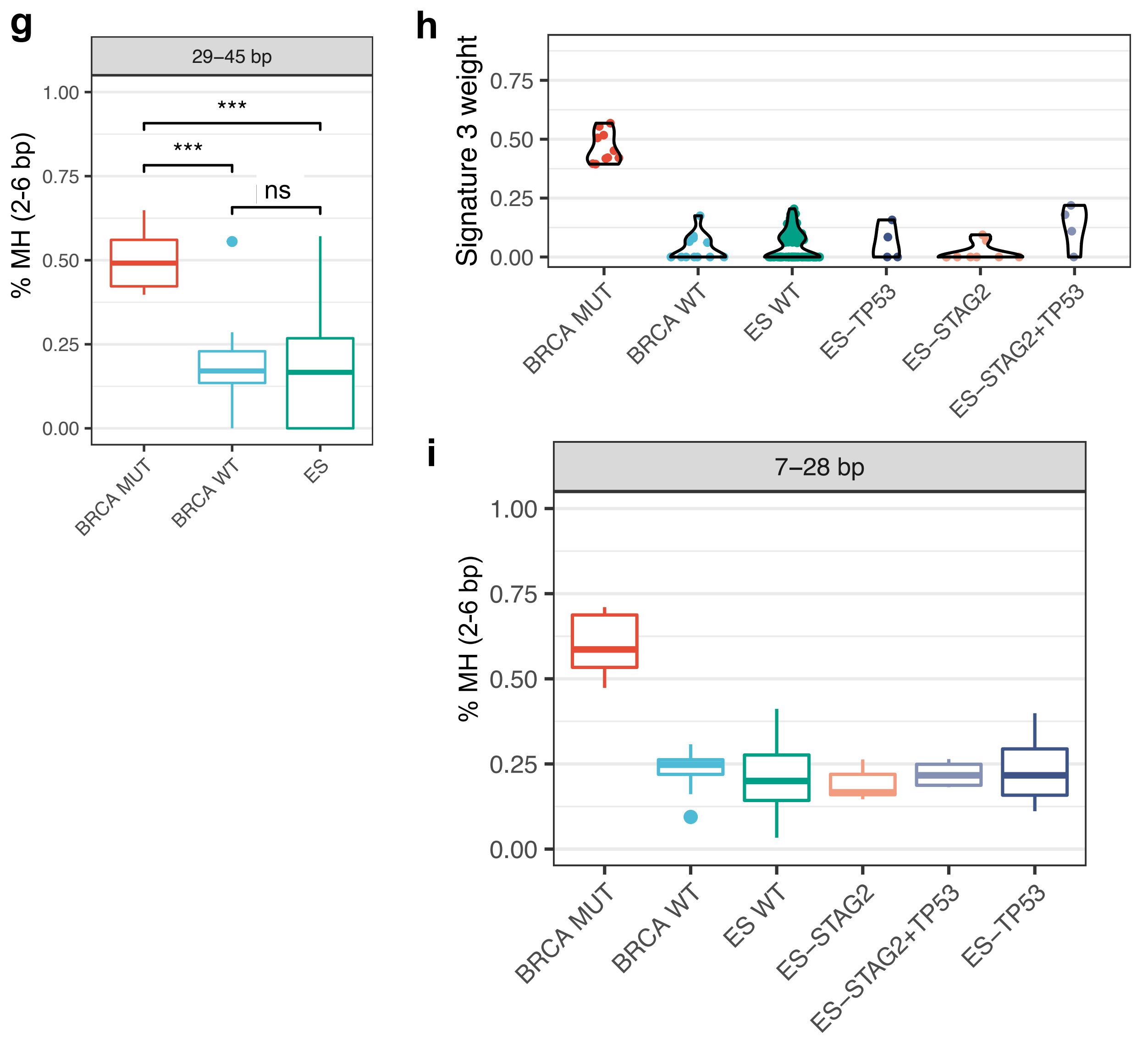

Supplemental Figure 2: ES patient tumors do not display the genomic scars of HR deficiency.
A) Mutational signature breakdown for BRCA mutant (MUT)/wildtype (WT) and ES tumors.
B) Total number of unique deletions per sample.
C) Deletion length per sample.
D) Deletion size per tumor sample shown individually for MuTect and Delly deletion calls.
E) Deletion size/microhomology (MH) profile for only BRCA WT and BRCA MUT samples showing separation between the groups present in the 7-28bp and 29-45bp bins in both the fraction of events in deletion size bin (X-axis) and percentage of MH (Y-axis).
F) Graph of Fisher test significance (p value) for deletion lengths and MH status.
G) Breakout of the 29-45 base pair (bp) deletion length bin showing the fraction of deletions with 2-6 bp microhomology.
H, I) Signature 3 weight (H) and 2-6bp breakpoint MH in the 7-28bp deletion bin (I) in ES samples subdivided by TP53 and STAG2 mutational status. ES WT are wildtype for p53 and STAG2.
For all panels, *** denotes p< 0.001 by Mann-Whitney test, ns denotes not significant.

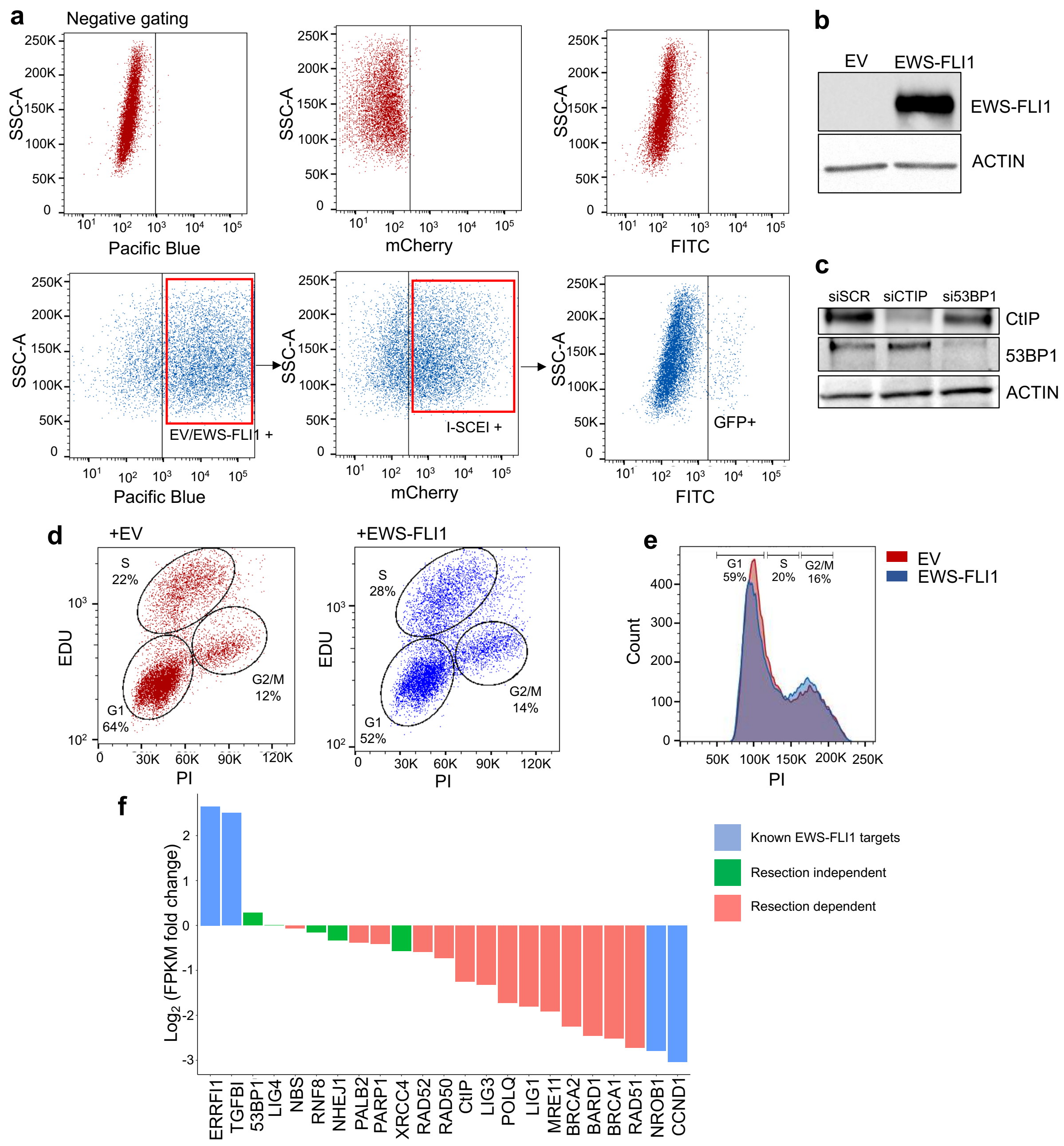

Supplemental Figure 3: EWS-FLI1 impairs resection-dependent DSB repair.
A) Gating schema for pathway-specific DSB repair reporter assays in U2OS cells using dual promoter vector expressing mTagBFP and EWS-FLI1 (or empty vector, EV), followed by introduction of mCherry-IScel endonuclease. GFP positivity is measured at 72 hours.
B) Representative Western blot showing EWS-FLI1 expression level in DSB repair reporter assays.
C) Western blot showing knockdown of relevant DSB repair genes.
D) Cell cycle profiles using EdU incorporation in U2OS cells +/- EWS-FLI1.
E) Cell cycle profiles using Propidium Iodide (PI) +/- EWS-FLI1.
F) Analysis of published RNAseq data[34] in A673 (ES cells) with EWS-FLI1 knockdown. Blue denotes canonical EWS-FLI1 targets, red/green denotes resection-dependent and independent DNA repair genes respectively.

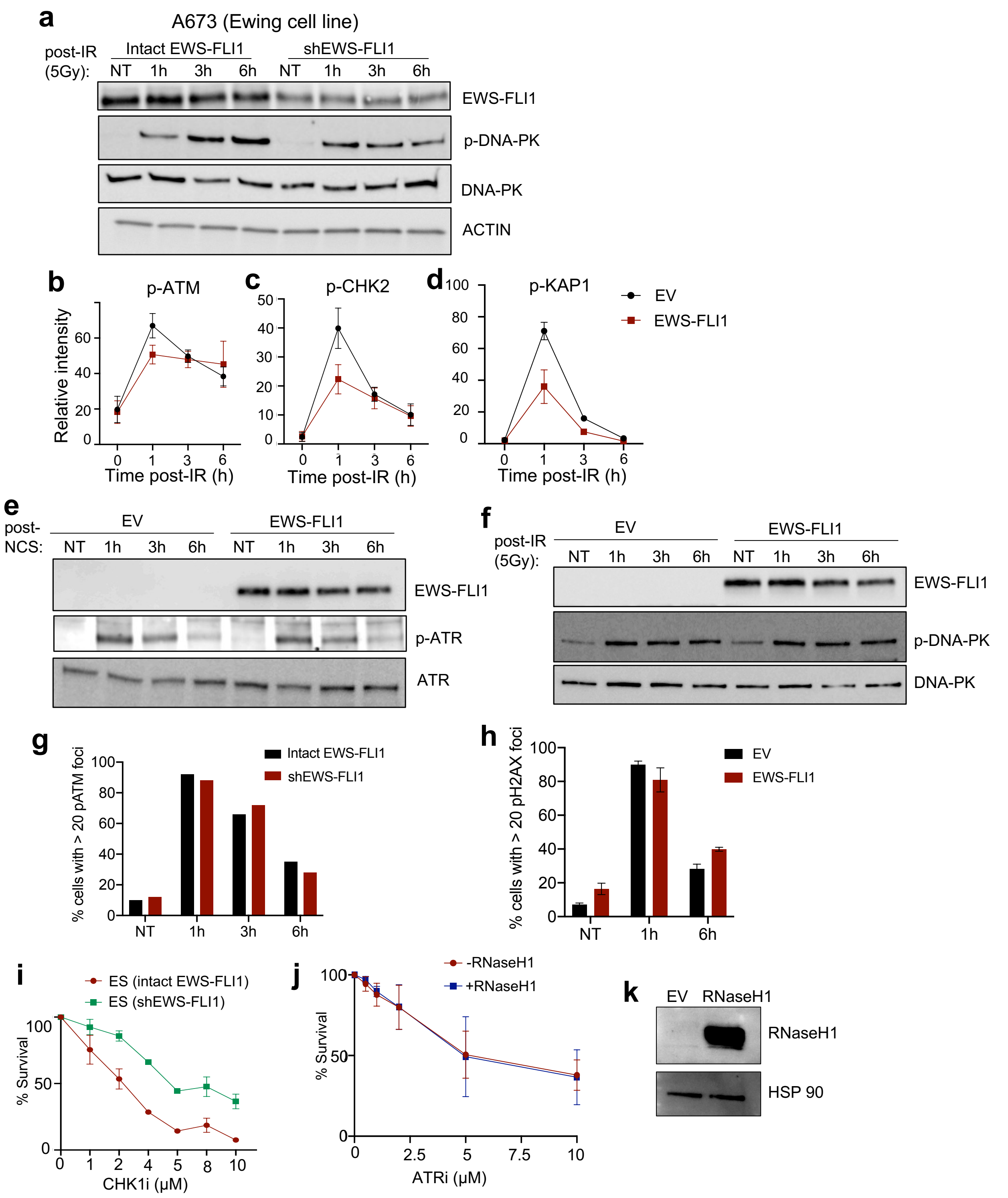

Supplemental Figure 4: ATM activation and signaling is defective in ES.

A) Western blotting upon 5 Gy IR at indicated time points in an ES cell line A673 with doxycycline(dox)-inducible shRNA against EWS-FLI1.

B-D) Quantification of Western blotting upon IR (5 Gy) at indicated time points in a non-ES cancer cell line (U2OS) upon expression of EV (empty vector) or EWS-FLI1.

E, F) Western blotting upon 5 Gy IR (D) or 200 ng/ml NCS treatment (E) at indicated time points in U2OS cells upon expression of EV or EWS-FLI1.
G) Quantification of p-ATM foci upon 5 Gy IR at indicated time points in ES cell line A673 with doxycycline(dox)-inducible shRNA against EWS-FLI1. n = 100 cells.
H) Quantification of pH2AX foci upon 5 Gy IR at indicated time points in non-ES cancer cell line (U2OS) upon EV or EWS-FLI1, n = 200 cells.
I) CHK1 inhibitor dose-response curves in A673 with dox-inducible shRNA against EWS-FLI1, $p<0.05$ by paired t-test, n=3.
J, K) ATR inhibitor (VE-821) dose-response curves for ES cell line A673 +/- RNAseH1 overexpression and confirmatory Western blot of RNAseH1 expression (K).
For all panels, error bars represent ± SEM and represent at least 3 replicates for each panel.

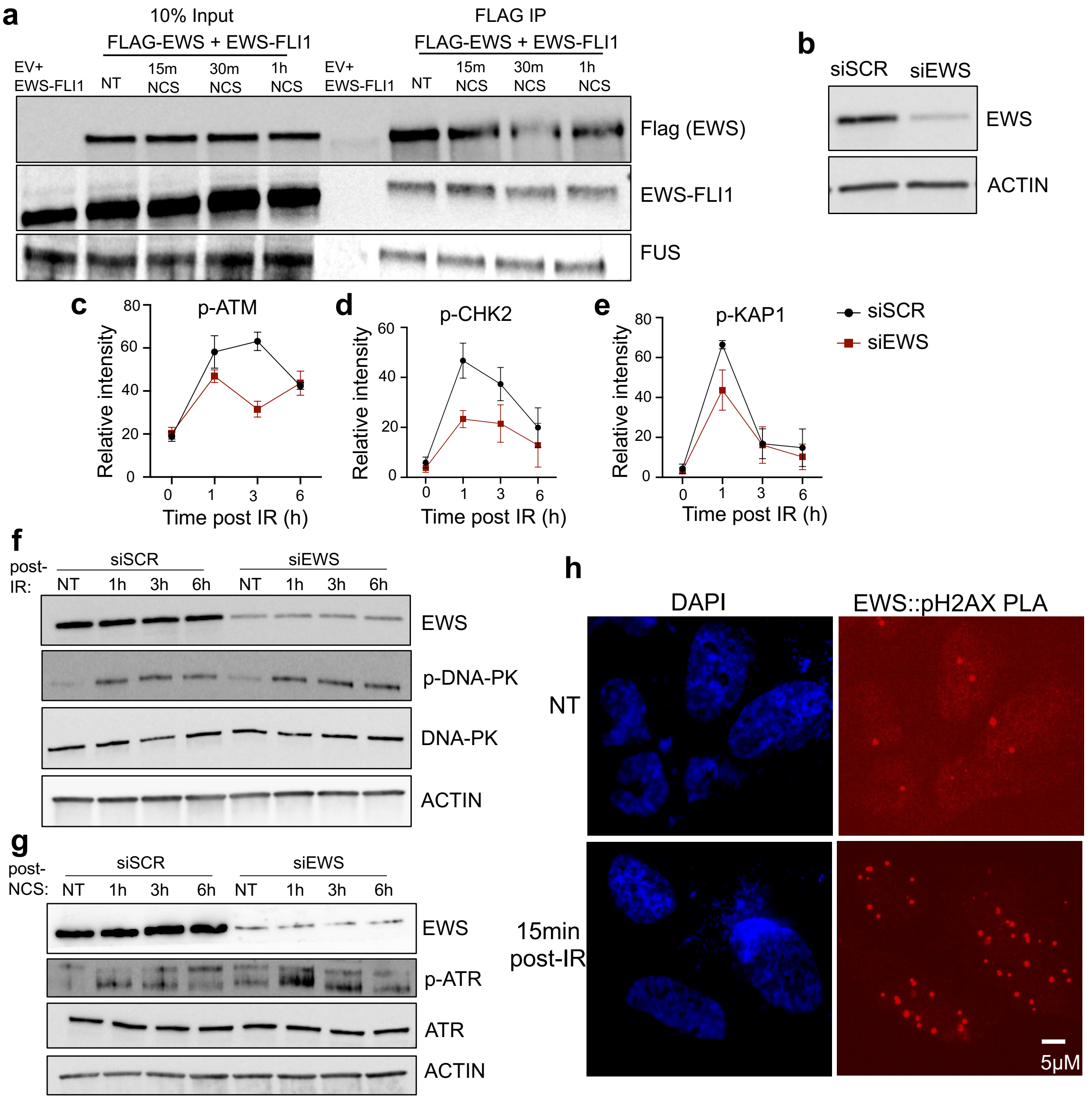

Supplementary Figure 5: Loss of native EWS phenocopies the DNA repair defects caused by EWS-FLI1.

A) Immunoprecipitation (IP) of FLAG-tagged EWS expressed in 293T cells +/- EWS-FLI1.
B) Representative Western blot of native EWS knockdown by siRNA in U2OS cells.
C-E) Quantification of Western blotting upon IR (5 Gy) at indicated time points in a non-ES cell line (U2OS) after 72-hour siRNA treatment against EWS or scramble (siSCR) control.
F, G) Representative Western blots upon 5 Gy IR (F) or 200 ng/ml NCS treatment (G) at indicated time points in a non-ES cell line (U2OS) after 72-hour siRNA treatment against EWS or scramble control.
For all panels, error bars represent ± SEM for at least 3 replicates for each panel.

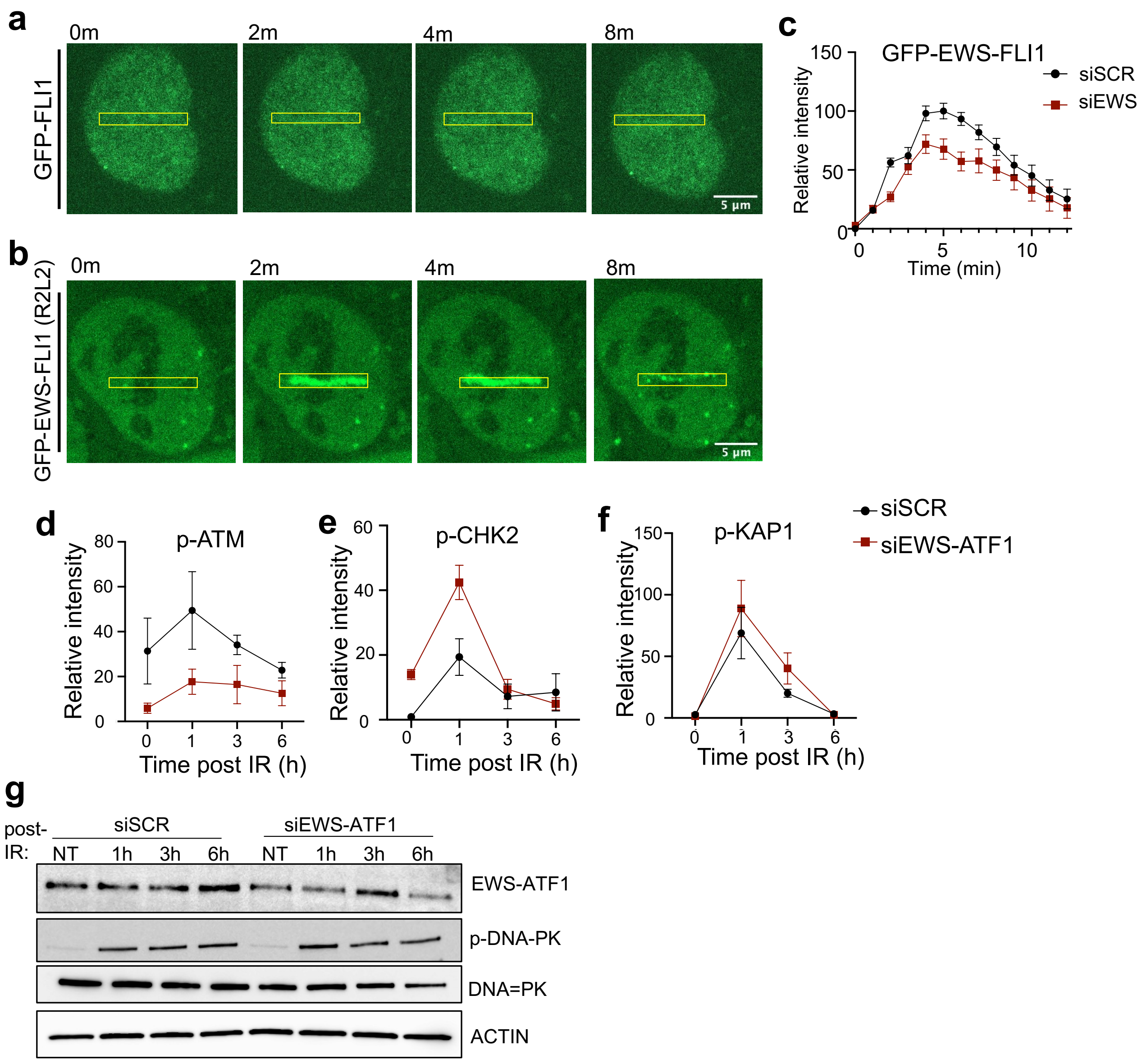

Supplementary Figure 6: FET fusion oncoproteins are recruited to DNA DSBs.
A) Laser micro-irradiation of full-length GFP-FLI1, yellow box denotes laser DSB stripe.
B) Laser micro-irradiation of GFP tagged DNA binding deficient mutant (R2L2) of EWS-FLI1.
C) Quantification of GFP-EWS-FLI1 accumulation at laser-induced DSBs in a non-ES cell line (U2OS) upon siRNA against native EWS or scramble (siSCR) for 72 hours. n=50 cells, $p<0.01$ using paired t-test.
D-F) Quantification of Western blotting upon IR (5Gy) at indicated time points in a CCS cell line (SU-CCS-1) after 72-hour siRNA treatment against EWS-ATF1 or scramble (siSCR) control.
G) Representative Western blots upon 5Gy IR at indicated time points in CCS cell line (SU-CCS-1) after 72-hour siRNA treatment against EWS-ATF1 or scramble (SCR) control.
For all panels, error bars represent ± SEM, at least 3 replicates for each panel.
